# Phenome-wide Mendelian randomization study evaluating the association of circulating vitamin D with complex diseases

**DOI:** 10.3389/fnut.2023.1108477

**Published:** 2023-03-29

**Authors:** Jin-jian Xu, Xiao-bin Zhang, Wen-tao Tong, Teng Ying, Ke-qi Liu

**Affiliations:** ^1^Guangdong Provincial Key Laboratory of Food, Nutrition and Health, Sun Yat-sen University (North Campus), Guangzhou, Guangdong, China; ^2^Department of Epidemiology, School of Public Health, Sun Yat-sen University (North Campus), Guangzhou, Guangdong, China; ^3^Department of Hepatobiliary Surgery, Jingdezhen No.1 People's Hospital, Jingdezhen, Jiangxi, China; ^4^Department of Cardiology, The First Affiliated Hospital of Jiangxi Medical College, Shangrao, Jiangxi, China; ^5^Department of Clinical Medicine, Jiangxi Medical College, Shangrao, Jiangxi, China

**Keywords:** circulating vitamin D, complex diseases, association, Mendelian randomization (MR) analysis, phenome wide association studies

## Abstract

**Background:**

Circulating vitamin D has been associated with multiple clinical diseases in observational studies, but the association was inconsistent due to the presence of confounders. We conducted a bidirectional Mendelian randomization (MR) study to explore the healthy atlas of vitamin D in many clinical traits and evaluate their causal association.

**Methods:**

Based on a large-scale genome-wide association study (GWAS), the single nucleotide polymorphism (SNPs) instruments of circulating 25-hydroxyvitamin D (25OHD) from 443,734 Europeans and the corresponding effects of 10 clinical diseases and 42 clinical traits in the European population were recruited to conduct a bidirectional two-sample Mendelian randomization study. Under the network of Mendelian randomization analysis, inverse-variance weighting (IVW), weighted median, weighted mode, and Mendelian randomization (MR)–Egger regression were performed to explore the causal effects and pleiotropy. Mendelian randomization pleiotropy RESidual Sum and Outlier (MR-PRESSO) was conducted to uncover and exclude pleiotropic SNPs.

**Results:**

The results revealed that genetically decreased vitamin D was inversely related to the estimated BMD (β = −0.029 g/cm^2^, *p* = 0.027), TC (β = −0.269 mmol/L, *p* = 0.006), TG (β = −0.208 mmol/L, *p* = 0.002), and pulse pressure (β = −0.241 mmHg, *p* = 0.043), while positively associated with lymphocyte count (β = 0.037%, *p* = 0.015). The results did not reveal any causal association of vitamin D with clinical diseases. On the contrary, genetically protected CKD was significantly associated with increased vitamin D (β = 0.056, *p* = 2.361 × 10^−26^).

**Conclusion:**

The putative causal effects of circulating vitamin D on estimated bone mass, plasma triglyceride, and total cholesterol were uncovered, but not on clinical diseases. Vitamin D may be linked to clinical disease by affecting health-related metabolic markers.

## Introduction

Vitamin D is an essential fat-soluble nutrition from cholecalciferol and steroid pro-hormone, which is predominately obtained from sunlight, dietary sources, and supplementation (1, 2). Vitamin D deficiency is common worldwide, with nearly one billion people experiencing vitamin D deficiency in 2019 (3). In this study, it is reported that 69.2% of the Asian population suffer from vitamin D deficiency, with 61% of postmenopausal women deficient in vitamin D , and the prevalence of vitamin D insufficiency among children is 56.2% (4). The deficiency in vitamin D is critical and is a contributor to the risk of metabolic diseases (5–7), cancers (8, 9), and all-cause mortality (10, 11).

Lowering 25-hydroxyvitamin D (25(OH)D) may act as a trigger for the inflammatory response to disturb the composition and function of circulating cytokines and growth factors (12), influencing endothelial cells (13), hence, increasing the risk of neurodegenerative disorders (14), cardiovascular disease (15, 16), musculoskeletal lesions (17), and mortality (11). The large-scale meta-analysis included 50 randomized controlled trials (RCTs) with a total of 74,655 participants and revealed that vitamin D supplementation statistically significantly reduced the risk of cancer death (RR: 0.85, 0.74–0.97) in adults compared with placebo (18). Moreover, vitamin D supplementation significantly reduced total cancer mortality in an updated meta-analysis of RCTs with 1,591 deaths (19). The meta-analysis comparing the highest with the lowest circulating 25(OH)D concentrations showed a 39% lower risk between levels of total 25(OH)D and colorectal cancer (CRC) risk (OR: 0.61, 0.52–0.71) with 11 case–control studies, and a 20% reduced CRC risk (HR: 0.80, 0.66–0.97) with six prospective cohort studies (20). Moreover, the effects of vitamin D supplementation on decreasing the LDL-c level (21) and releasing insulin resistance (22) were uncovered.

Inversely, the results from an RCT of vitamin D3 (cholecalciferol) at a dose of 2,000 IU per day during a median follow-up of 5.3 years found that supplementation with vitamin D3 did not result in a lower incidence of invasive cancer or cardiovascular events than placebo (23). The negative results were observed in another RCT with 5,108 older adult participants (65.9 years) during a median follow-up of 3.3 years, in which monthly high-dose vitamin D3 (200,000 IU) supplementation did not prevent CVD (24). Meanwhile, the studies did not illuminate the benefits of vitamin D3 supplementation for relapse-free survival of digestive tract cancer at 5 years (25, 26). Although numerous studies explored the association of vitamin D with many clinical diseases, inconsistent results were produced, and the potential confounders may contribute to the unobvious benefit of vitamin D.

Recently, single nucleotide polymorphism (SNP) that was identified by genome-wide association studies (GWASs) was recruited as an instrument in Mendelian randomization (MR) analyses to explore the causal effects of exposures on outcomes (6, 27–30). The MR analysis demonstrated that genetically decreased 25(OH)D was associated with the risk of multiple sclerosis (MS) and provided strong evidence for the causal role of vitamin D in MS susceptibility (8). Moreover, a significant association between 25(OH)D levels and T2DM was found in a European-descent MR with SNP instruments of vitamin D synthesis (31). Inversely, no evidence for the effects of 25(OH)D on T2DM was observed in another MR study with four instrumental SNPs of 25(OH)D concentrations (27). Meanwhile, the delicate associations between genetically predicted 25(OH)D and multiple outcomes were observed, such as in inflammatory bowel disease (IBD) (28), bone mineral density (BMD) (32), non-alcoholic fatty liver disease (NAFLD) (29), and hypertension (33). The genetic instruments of 25(OH)D recruited in a previous MR analysis were limited to four or six variants involved in vitamin D synthesis (DHCR7/NADSYN1, rs12785878 and CYP2R1, rs10741657), transportation (GC, rs3755967), and degradation (CYP24A1, rs17216707), as well as two novel vitamin D metabolism pathways, such as SEC23A (Sec23 homolog A, coat protein complex II component, rs8018720) and AMDHD1 (amidohydrolase domain containing 1, rs10745742). The overall estimate of heritability of 25(OH)D attributable to statistically significant GWAS common SNPs was 2.84% (34). The limited efficiency of SNP instruments may lead to inconsistent results.

In the current study, we conducted a systematic literature review of previous MR studies on 25(OH)D and performed a bidirectional MR analysis to examine the causal association between genetic determinants of 25(OH)D and cardiovascular diseases (CAD), fracture, rheumatoid arthritis (RA), Alzheimer's dementia (AD), inflammatory bowel disease (IBD), chronic kidney disease (CKD), and related clinical risk factors using summary data from large-scale genome-wide association studies.

## Methods

### Study design

The GWAS summary statistics about 10 clinical diseases (including chronic kidney disease, stroke, type-2 diabetes, coronary artery disease, CAD with diabetes, Crohn's disease, ulcerative colitis, fracture, rheumatoid arthritis, and Alzheimer's dementia) and 42 clinical traits (including the anthropometric index, glycemic traits, serum lipids, cardiovascular measurements, kidney function, musculoskeletal health, bone mineral density, and blood cell and plasma cytokine) were collected from publicly available data sources and analyzed to estimate the genetic causal relationship of serum 25(OH)D with multifarious clinical outcomes through bidirectional two-sample Mendelian randomization (MR) (35). The MR procedure consists of three assumptions (36): (1) the genetic instrumental variables are strongly associated with exposures; (2) the genetic instrumental variables are not associated with any known or unmeasured confounders: influencing the association between genetic variants and outcomes; (3) the genetic variants are associated with outcomes only through exposures: variants causing significant effects on outcomes not through other pathways, no horizontal pleiotropy. The framework of the MR study is presented in Supplementary Figure 1. Ethics approval was not required for this study.

### Identification of SNPs associated with 25(OH)D and clinical outcomes

Based on the large-scale GWAS, which consisted of 443,734 participants from the UK Biobank, the 138 conditionally independent SNPs for 25(OH)D, mapped to 69 distinct loci, among which 63 were previously not reported, were selected at the genome-wide significance level (6.6 × 10^−9^) after adjusting for age, sex, and season of 25(OH)D measurement (37). Of these conditionally independent SNPs, 53 (38%) had a minor allele frequency (MAF) of <5%, and 85 (62%) were common (MAF ≥ 5%). The 53 SNPs with a MAF of <5% conferred an average absolute effect of 0.23 standard deviations on standardized log transformed 25(OH)D levels per effect allele, compared to 0.03 standard deviations of the 85 SNPs with a MAF of ≥5%. The average absolute effect on 25(OH)D of the 53 low frequency and rare variants was at least 7 times larger than the average effect of the 85 common SNPs. The known vitamin D loci (*CYP2R1, DHCR7, GC, CYP24A1, AMDHD1*, and *SEC23A*) were replicated in the study (Supplementary Table 1; Supplementary Figure 2). Serum 25(OH)D in this study was measured by liquid chromatography-tandem mass spectrometry.

The characteristics of selected instrumental SNPs for specific clinical diseases and clinical traits are presented in Table 1. Contributing studies received ethical approval from their respective institutional review boards. Informed consent was obtained from all participants of contributing studies. The GWAS summary statistics of 10 clinical diseases (Supplementary Tables 2, 3) and 42 clinical traits (Supplementary Table 4) were collected from publicly available resources. We conducted a comprehensive literature review to test the horizontal pleiotropy in selected SNPs and evaluate whether any of the SNPs were influenced by linkage disequilibrium (LD). To examine assumptions 2 and 3, we chose the variant with the lowest *p*-value for association with clinical outcomes.

**Table 1:** Description of selected clinical outcomes.

**Disease or trait**	**Sample size**	**Population**	**Data sources**	**Years**
25(OH)D (37)	401,460	European	UK BioBank	2020
**Anthropometric index**				
BMI (38)	526,508	Transethnic	GRASP	2018
Waist-to-hip ratio (39)	694,649	European	GIANT and UK BioBank	2019
Obesity (40)	276,007	European	GIANT	2018
**Glycemic traits**				
Fasting glucose (41)	133,010	European	MAGIC	2012
Fasting insulin (41)	133,010	European	MAGIC	2012
Fasting proinsulin (42)	16,378	European	MAGIC	2011
HbA1c (43)	46,368	European	MAGIC	2010
**Lipids**				
HDL-C (44)	188,578	Transethnic	GLGC	2013
LDL-C (44)	188,578	Transethnic	GLGC	2013
Total cholesterol (44)	188,578	Transethnic	GLGC	2013
Triglycerides (44)	188,578	Transethnic	GLGC	2013
**Cardiovascular measurements**				
PP (45)	1,006,863	Transethnic	GRASP	2018
SBP (5‘9)	1,006,863	Transethnic	GRASP	2018
DBP (45)	1,006,863	Transethnic	GRASP	2018
Hypertension (46)	327,288	Transethnic	GRASP	2018
Heart rate variability (47)	53,174	European	GRASP	2017
**Kidney function**				
eGFR (48)	765,348	Transethnic	CKDGen	2019
UAUCr (49)	327,616	European	GRASP	2019
UKUCr (49)	327,616	European	GRASP	2019
UNaUCr (49)	327,616	European	GRASP	2019
UNaUK (49)	327,616	European	GRASP	2019
**Musculoskeletal health**				
Lean body mass (50)	85,519	European	GEFOS	2017
Grip strength (51)	195,180	European	GRASP	2017
Gait speed (52)	34,066	European	GRASP	2017
**Bone mineral density**				
eBMD (53)	488,683	European	GEFOS	2017
Total body BMD (54)	666,628	Transethnic	GEFOS	2018
Forearm BMD (55)	32,965	European	GEFOS	2015
Femoral neck BMD (55)	32,965	European	GEFOS	2015
Lumbar spine BMD (55)	32,965	European	GEFOS	2015
**Blood cell and plasma cytokine**				
Platelet count (56)	173,480	European	GRASP	2016
Lymphocyte count (56)	173,480	European	GRASP	2016
Red blood cell count (56)	173,480	European	GRASP	2016
White blood cell count (56)	173,480	European	GRASP	2016
Interleukin-1-beta (57)	8,293	European	GRASP	2017
Interleukin-6 (57)	8,293	European	GRASP	2017
Interleukin-7 (57)	8,293	European	GRASP	2017
Interleukin-8 (57)	8,293	European	GRASP	2017
Interleukin-9 (57)	8,293	European	GRASP	2017
Interleukin-10 (57)	8,293	European	GRASP	2017
Beta nerve growth factor (57)	8,293	European	GRASP	2017
Tumor necrosis factor-alpha (57)	8,293	European	GRASP	2017
Vascular endothelial growth factor (57)	8,293	European	GRASP	2017
**Clinical disease**				
Chronic kidney disease (48)	625,219 (64,164 cases)	European	CKDGen	2019
Stroke (58)	521,612 (67,162 cases)	European	ISGC	2018
Type 2 diabetes (59)	898,130 (74,124 cases)	European	DIAGRAM	2018
Coronary artery disease (60)	184,305 (60,801 cases)	Transethnic	CARDIoGRAMplusC4D	2018
CAD with diabetes (61)	15,666 (3,968 cases)	European	GRASP	2015
Crohn's disease (62)	51,109 (22,027 cases)	European	IBDGenetics	2010
Ulcerative colitis (63)	48,950 (16,315 cases)	European	IBDGenetics	2011
Fracture (64)	264,973 (37,857 cases)	Transethnic	GEFOS	2018
Rheumatoid arthritis (65)	103,638 (29,880 cases)	Transethnic	GRASP	2014
Alzheimer's dementia (66)	455,258 (71,880 cases)	Transethnic	GRASP	2019

GRASP, genome-wide repository of associations between SNPs and phenotypes; GIANT, genetic investigation of anthropometric traits; MAGIC, The Meta-Analyses of Glucose and Insulin-Related Traits Consortium; GLGC, The Global Lipids Genetics Consortium; CKDGen, The Chronic Kidney Disease Consortium; GEFOS, The GEnetic Factors for OSteoporosis consortium; ISGC, International Stroke Genetics Consortium; DIAGRAM, diabetes genetics replication and meta-analysis; CARDIoGRAMplusC4D, coronary artery disease genome-wide replication and meta-analysis plus the coronary artery disease (C4D) genetics; IBDGenetics, International Inflammatory Bowel Disease Genetics Consortium; BMI, body mass index; eBMD, estimated bone mineral density; HDL, high density lipoprotein cholesterol; LDL, low density lipoprotein cholesterol; PP, pulse pressure; SBP, systolic blood pressure; DBP, diastolic blood pressure; eGFR, estimated glomerular filtration rate; UAUCr, urinary albumin to creatinine ratio; UKUCr, urinary potassium to creatinine ratio; UNaUCr, urinary sodium to creatinine ratio; UNaUK, urinary sodium to potassium ratio.

### Characteristics of selected data sources

To comprehensively explore the effective atlas of vitamin D on numerous healthy outcomes (clinical diseases and clinical traits), the specific genetic SNPs were selected from public sources (Table 1). For clinical diseases, the GWAS summary-level data were extracted from the Chronic Kidney Disease Consortium (CKDGen; 561,055 controls and 64,164 cases) for CKD; the Diabetes Genetics Replication and Meta-analysis (DIAGRAM) Consortium for T2DM (824,006 controls and 74,124 cases); the International Stroke Genetics Consortium (ISGC) for stroke (454,450 controls and 67,162 cases); the coronary artery disease genome-wide replication and meta-analysis (CARDIoGRAM) plus the Coronary Artery Disease (C4D) Genetics (CARDIoGRAMplusC4D) Consortium for CAD (123,504 controls and 60,801 cases), and CAD with diabetes (11,698 controls and 3,968 cases); the International Inflammatory Bowel Disease Genetics Consortium (IIBDGC) for IBD (48,950 for ulcerative colitis and 51,109 for Crohn's disease); the Psychiatric Genomics Consortium (PGC-ALZ), the Alzheimer's Disease Sequencing Project (ADSP), and the International Genomics of Alzheimer's Project (IGAP) for AD (383,378 controls and 71,880 cases); the GEnetic Factors for OSteoporosis consortium (GEFOS) for fracture (227,116 controls and 37,857 cases); and Genetics and Allied research in Rheumatic diseases Networking (GARNET) and Rheumatoid Arthritis Consortium International (RACI) for RA (73,758 controls and 29,880 cases).

For clinical traits, 20 GWAS summary datasets for genetic determinants were available. Genome-wide association analyses have been published for adiposity (BMI, WHR adjusted BMI, and obesity) by the Genetic Investigation of Anthropometric Traits (GIANT) Consortium; glycemic traits [hemoglobin A1c (HbA1c), fasting glucose, fasting insulin, and fasting proinsulin] by the Meta-Analyses of Glucose and Insulin-Related Traits Consortium (MAGIC), plasma lipids (HDL-C, LDL-C, TC, and TG) by the Global Lipids Genetics Consortium (GLGC), bone mineral density (estimated BMD, total body BMD, forearm, femoral neck, and lumbar spine BMD), musculoskeletal measurements (lean body mass, hand grip strength, and gait speed), blood pressure (systolic BP, diastolic BP, and pulse pressure), and heart rate variability by the GEFOS and the genome-wide repository of associations between SNPs and phenotypes (GRASP). Meanwhile, the circulating cytokines and growth factors for inflammation and urinary biomarkers for kidney function, such as the urinary albumin to creatinine ratio (UACR), the urinary potassium to creatinine ratio (UK/UCr), the urinary sodium to creatinine ratio (UNa/UCr), and the urinary sodium to potassium ratio (UNa/UK), were explored.

The definitions of clinical disease and clinical traits are explained in Supplementary material. All clinical diseases were diagnosed using ICD-10 (international classification of diseases, 10th revisions) codes. The ICD-10 codes of chronic kidney disease, stroke, type-2 diabetes, coronary artery disease, CAD with diabetes, Crohn's disease, ulcerative colitis, fracture, rheumatoid arthritis, and Alzheimer's dementia are presented in Supplementary Table 7.

### Statistical analysis

The summary datasets consist of effect sizes and standard errors of outcomes and exposures. However, the effect/non-effect alleles must be harmonized between outcome and exposure, when the effect allele was flipped (effect/non-effect alleles were G/T for the exposure and T/G for the outcome). Meanwhile, the alleles of outcome were matched with exposure alleles and effect alleles were aligned, when the strand of SNPs was flipped (the effect/non-effect alleles were G/T for the exposure and C/A for the outcome). Finally, we eliminated the incompatible SNPs (effect/non-effect alleles were A/G for the exposure and A/T for the outcome).

All the SNPs that independently (linkage disequilibrium *r*^2^ < 0.01) and strongly associated with the exposures at the genome-wide significant levels were extracted to verify the horizontal pleiotropy. The associations of curated SNPs with traits were assessed online (GWAS Catalog, https://www.ebi.ac.uk/gwas; ClinicalTrials. gov, https://clinicaltrials.gov; PhenoScanner, http://www.phenoscanner.medschl.cam.ac.uk) through Mendelian randomization analysis. Moreover, the Mendelian Randomization Pleiotropy RESidual Sum and Outlier (MR-PRESSO) was employed to identify and remove pleiotropic SNPs by assessing outliers among the included SNPs contributing to the Mendelian randomization estimate. We repeated the analyses after excluding potentially pleiotropic SNPs. Then, inverse-variance weighting (IVW), weighted median, weighted mode, and Mendelian randomization (MR)–Egger regression were applied to explore the causal effect of genetically decreased vitamin D on a wide spectrum of phenotypes (Supplementary material). For sensitivity analysis, bidirectional Mendelian randomization was conducted to validate the causal effects of genetic determinants of clinical diseases on vitamin D. All analyses were performed using R Version 4.0.2 with the R package Mendelian Randomization, MR-PRESSO.

## Results

### Associations of genetic 25(OH)D with the risk of clinical diseases

**Figure 1:**
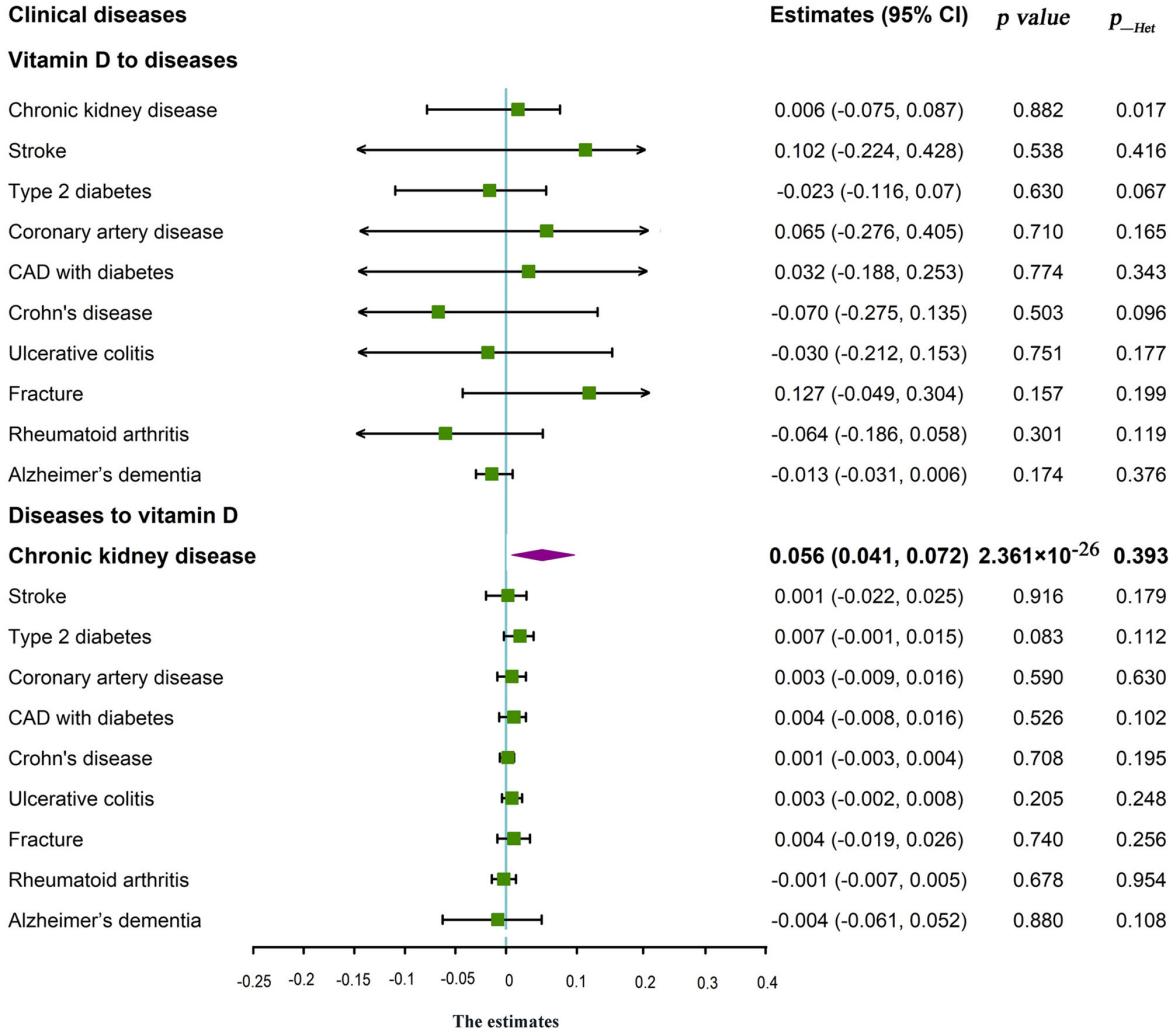
Forest plot of the association between circulating 25(OH)D and clinical diseases in bidirectional Mendelian randomization analysis. CAD, coronary artery disease; *p*_Het, heterogeneity statistics.

The results failed to reveal any effect of 25(OH)D on many clinical diseases (All *p* > 0.05) in IVW analysis (Table 2, Figure 1). Similar results were obtained in the weighted median and weighted mode statistics (*p* > 0.05; Supplementary Table 5). There was no significant horizontal pleiotropy and heterogeneity in all selected SNPs after excluded pleiotropic variants by MR-PRESSO (*p*__Het_ > 0.05, intercept *p* > 0.05, global test *p* > 0.05; Table 2, Figure 1, Supplementary Table 5, Supplementary Figures 3a–j).

**Table 2:** Association of vitamin D with clinical diseases using inverse-variance weighting Mendelian randomization study.

**Clinical disease**	***N*** **SNPs**	**IVW**		
		**Estimates (95% CI)**	***p*** **-value**	***p*** **_Het**
**Vitamin D to diseases**				
Chronic kidney disease	75	0.006 (−0.075 to 0.087)	0.882	0.017
Stroke	34	0.102 (−0.224 to 0.428)	0.538	0.416
Type 2 diabetes	70	−0.023 (−0.116 to 0.07)	0.630	0.067
Coronary artery disease	29	0.065 (−0.276 to 0.405)	0.710	0.165
CAD with diabetes	76	0.032 (−0.188 to 0.253)	0.774	0.343
Crohn's disease	81	−0.070 (−0.275 to 0.135)	0.503	0.096
Ulcerative colitis	85	−0.030 (−0.212 to 0.153)	0.751	0.177
Fracture	33	0.127 (−0.049 to 0.304)	0.157	0.199
Rheumatoid arthritis	66	−0.064 (−0.186 to 0.058)	0.301	0.119
Alzheimer's dementia	95	−0.013 (−0.031 to 0.006)	0.174	0.376
**Diseases to vitamin D**				
Chronic kidney disease	17	0.056 (0.041 to 0.072)	2.361 × 10^−26^	0.393
Stroke	13	0.001 (−0.022 to 0.025)	0.916	0.179
Type 2 diabetes	28	0.007 (−0.001 to 0.015)	0.083	0.112
Coronary artery disease	25	0.003 (−0.009 to 0.016)	0.590	0.630
CAD with diabetes	46	0.004 (−0.008 to 0.016)	0.526	0.102
Crohn's disease	60	0.001 (−0.003 to 0.004)	0.708	0.195
Ulcerative colitis	37	0.003 (−0.002 to 0.008)	0.205	0.248
Fracture	14	0.004 (−0.019 to 0.026)	0.740	0.256
Rheumatoid arthritis	26	−0.001 (−0.007 to 0.005)	0.678	0.954
Alzheimer's dementia	26	−0.004 (−0.061 to 0.052)	0.880	0.108

SNPs, single nucleotide polymorphisms; CAD, coronary artery disease; IVW, inverse-variance weighting; p_Het, heterogeneity statistics.

The inversed association between increased risk of CKD with 25(OH)D (β = 0.056, 95% CI: 0.04–0.072, *p* = 2.361 × 10^−26^, *p*__Het_ = 0.393) was found in the IVW analysis (Table 2, Figure 1). Similarly, significant associations were found in the weighted median (β = 0.057, 95% CI: 0.035–0.080, *p* = 3.128 × 10^−25^) and weighted mode (β = 0.056, 95% CI: 0.021–0.091, *p* = 0.002). There was no heterogeneity and horizontal pleiotropy with MR–Egger regression (Intercept *p* = 0.335) after excluded pleiotropic variants using the restrictive MR pleiotropy residual sum and outlier test (MR-PRESSO) method (Global test *p* = 0.469; Supplementary Table 5). In addition, no evidence supported the effects of clinical diseases on 25(OH)D, except CKD in reversed MR analysis (Table 2, Figure 1, Supplementary Table 5, Supplementary Figures 4a–j).

### Association of genetic 25(OH)D with clinical traits

**Figure 2:**
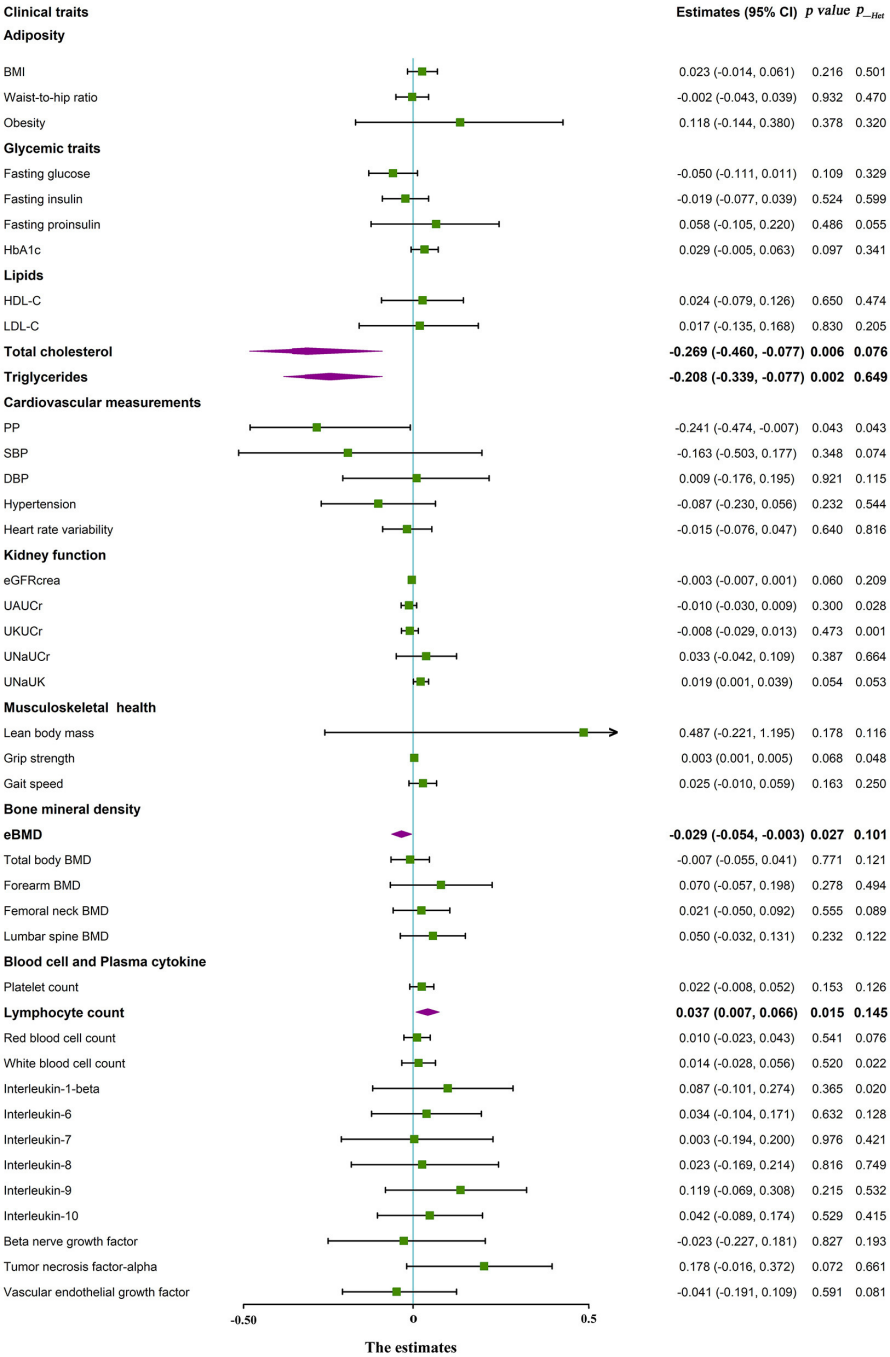
Forest plot about the association between circulating 25(OH)D and clinical traits in Mendelian randomization analysis. BMI, body mass index; eBMD, estimated bone mineral density; HDL, high density lipoprotein cholesterol; LDL, low density lipoprotein cholesterol; PP, pulse pressure; SBP, systolic blood pressure; DBP, diastolic blood pressure; eGFR, estimated glomerular filtration rate; UAUCr, urinary albumin to creatinine ratio; UKUCr, urinary potassium to creatinine ratio; UNaUCr, urinary sodium to creatinine ratio; UNaUK, urinary sodium to potassium ratio; *p*___Het, heterogeneity statistics.

Mendelian randomization analyses were conducted to assess the association of plasma 25(OH)D with 42 clinical traits. The results revealed that genetically decreased 25(OH)D was strongly correlated with estimated BMD (g/cm^2^), TC (mmol/L), TG (mmol/L), and PP (mmHg), and negatively associated with lymphocyte count (%; All *p* < 0.05, *p*_Het > 0.05) in IVW (Table 3, Figure 2). There was no evidence for the association of genetically decreased vitamin D with the anthropometric index, glycemic traits, kidney function, and musculoskeletal health (Table 3, Figure 2, Supplementary Table 6). The intercepts in MR–Egger test were tightly centered around the null, which revealed the statistic effects of genetic instruments in Mendelian randomization analyses did not be influenced by pleiotropy.

**Table 3:** Association of vitamin D with clinical traits using inverse-variance weighting Mendelian randomization.

**Clinical traits**	***N*** **SNPs**	**IVW**		
		**Estimates (95% CI)**	***p*** **-value**	***p*** **_Het**
**Adiposity**				
BMI	23	0.023 (−0.014 to 0.061)	0.216	0.501
Waist-to-hip ratio	23	−0.002 (−0.043 to 0.039)	0.932	0.470
Obesity	33	0.118 (−0.144 to 0.380)	0.378	0.320
**Glycemic traits**				
Fasting glucose	38	−0.05 (−0.111 to 0.011)	0.109	0.329
Fasting insulin	38	−0.019 (−0.077 to 0.039)	0.524	0.599
Fasting proinsulin	35	0.058 (−0.105 to 0.220)	0.486	0.055
HbA1c	36	0.029 (−0.005 to 0.063)	0.097	0.341
**Lipids**				
HDL-c	27	0.024 (−0.079 to 0.126)	0.650	0.474
LDL-c	23	0.017 (−0.135 to 0.168)	0.830	0.205
Total cholesterol	18	−0.269 (−0.46 to −0.077)	0.006	0.076
Triglycerides	22	−0.208 (−0.339 to −0.077)	0.002	0.649
**Cardiovascular measurements**				
PP	45	−0.241 (−0.474 to −0.007)	0.043	0.043
SBP	41	−0.163 (−0.503 to 0.177)	0.348	0.074
DBP	47	0.009 (−0.176 to 0.195)	0.921	0.115
Hypertension	9	−0.087 (−0.23 to 0.056)	0.232	0.544
Heart rate variability	36	−0.015 (−0.076 to 0.047)	0.640	0.816
**Kidney function**				
eGFRcrea	50	−0.003 (−0.007 to 0.001)	0.060	0.209
UAUCr	85	−0.01 (−0.030 to 0.009)	0.300	0.028
UKUCr	85	−0.008 (−0.029 to 0.013)	0.473	0.001
UNaUCr	13	0.033 (−0.042 to 0.109)	0.387	0.664
UNaUK	91	0.019 (0.001 to 0.039)	0.054	0.053
**Musculoskeletal health**				
Lean body mass	37	0.487 (−0.221 to 1.195)	0.178	0.116
Grip strength	70	0.003 (0.001 to 0.005)	0.068	0.048
Gait speed	34	0.025 (−0.01 to 0.059)	0.163	0.250
**Bone mineral density**				
eBMD	11	−0.029 (−0.054 to −0.003)	0.027	0.101
Total body BMD	10	−0.007 (−0.055 to 0.041)	0.771	0.121
Forearm BMD	74	0.07 (−0.057 to 0.198)	0.278	0.494
Femoral neck BMD	68	0.021 (−0.05 to 0.092)	0.555	0.089
Lumbar spine BMD	66	0.05 (−0.032 to 0.131)	0.232	0.122
**Blood cell and plasma cytokine**				
Platelet count	96	0.022 (−0.008 to 0.052)	0.153	0.126
Lymphocyte count	95	0.037 (0.007 to 0.066)	0.015	0.145
Red blood cell count	86	0.01 (−0.023 to 0.043)	0.541	0.076
White blood cell count	90	0.014 (−0.028 to 0.056)	0.520	0.023
Interleukin-1-beta	76	0.087 (−0.101 to 0.274)	0.365	0.020
Interleukin-6	79	0.034 (−0.104 to 0.171)	0.632	0.128
Interleukin-7	79	0.003 (−0.194 to 0.2)	0.976	0.421
Interleukin-8	79	0.023 (−0.169 to 0.214)	0.816	0.749
Interleukin-9	79	0.119 (−0.069 to 0.308)	0.215	0.532
Interleukin-10	79	0.042 (−0.089 to 0.174)	0.529	0.415
Beta nerve growth factor	79	−0.023 (−0.227 to 0.181)	0.827	0.193
Tumor necrosis factor-alpha	79	0.178 (−0.016 to 0.372)	0.072	0.661
Vascular endothelial growth factor	79	−0.041 (−0.191 to 0.109)	0.591	0.081

SNPs, single nucleotide polymorphisms; CAD, coronary artery disease; BMI, body mass index; eBMD, estimated bone mineral density; HDL, high-density lipoprotein cholesterol; LDL, low-density lipoprotein cholesterol; PP, pulse pressure; SBP, systolic blood pressure; DBP, diastolic blood pressure; eGFR, estimated glomerular filtration rate; UAUCr, urinary albumin to creatinine ratio; UKUCr, urinary potassium to creatinine ratio; UNaUCr, urinary sodium to creatinine ratio; UNaUK, urinary sodium to potassium ratio; IVW, inverse-variance weighting; p_Het, heterogeneity statistics.

## Discussion

In the present Mendelian randomization (MR) study, our findings did not support the putative causal effects of 25(OH)D on multiple clinical diseases. Genetically decreased 25(OH)D was significantly associated with the estimated bone mineral density (eBMD), plasma cholesterol, pulse pressure, and elevated lymphocyte count. A bidirectional MR study did not reveal the significant effects of CVD, IBD, T2DM, AD, and musculoskeletal disorders on 25(OH)D concentration. However, chronic kidney disease was positively associated with decreased 25(OH)D.

Dietary and skin-derived vitamin D will play biological roles in multiple organs after hydroxylating in the liver and kidney (67). Patients with chronic kidney disease will decrease the biosynthesis of circulating 25(OH)D by reducing the production of hydroxylase (68). Vitamin D exerts a biological role by binding the vitamin D receptor (VDR) (69), which is commonly found in musculoskeletal cells and various extracellular tissues, such as parathyroid tissue, intestinal tissue, and kidneys (70). Vitamin D deficiency not only increased the risk of rickets, osteomalacia, and osteoporotic fractures (71) but also contributed to extra-skeletal disorders (72) in epidemiological studies. However, no significant associations of 25(OH)D with the risk of clinical diseases were found in our study. Meanwhile, the reversed MR analysis did not reveal any impact of multiple chronic diseases on serum 25(OH)D, except for CKD. The relationship between vitamin D and clinical disease may not be linear, and a linear analysis may yield negative results. A non-linear association of 25(OH)D deficiency with the risk of cardiovascular disease was discovered in a non-linear MR analysis (73), and the non-linear dose–response relationships between 25(OH)D concentrations and coronary heart disease, stroke, and mortality outcomes were discovered in a stratified MR analysis (74).

A weak correlation of 25(OH)D with estimated BMD of heel was found, but not with femoral neck BMD (FN-BMD) or lumbar spine BMD (LS-BMD) in our study. Meta-analysis of RCTs revealed that vitamin D supplementation was not associated with a lower risk of fractures in older adults (75). The MR study showed that the SNP instruments (rs2282679, rs117913124, rs10741657, rs12785878, and rs727479) were not associated with femoral neck BMD (FN-BMD), lumbar spine BMD (LS-BMD), or estimated BMD (eBMD), but rs6013897 near CYP24A1 was associated with FN-BMD at borderline statistical significance (*p* = 0.01). High 25(OH)D concentration was not associated with higher FN-BMD (*p* = 0.37) or LS-BMD (*p* = 0.49) in the inverse-variance weighted analysis or in sensitivity analyses but was associated with estimated BMD (*p* = 0.02) (76). The differences in the biological pathways of 25(OH)D-SNPs may be the critical factor causing the contradictory result. Mendelian randomization analyses that combine all SNP effects may mask the role of individual SNPs. Moreover, the beneficial effects of vitamin D on many diseases may be largely due to undetected confounders in an epidemiologic study.

In the present study, no significant associations of genetic 25(OH)D with the risk of CAD, stroke, and T2DM were uncovered. The putative causal effects of circulating vitamin D on plasma triglyceride and total cholesterol were uncovered. Previous observational studies revealed that circulating 25(OH)D was inversely associated with blood pressure and the risk of type-2 diabetes (77), but positively correlated with blood lipids (triglycerides, HDL-c, LDL-c, and total cholesterol) (16). Moreover, vitamin D supplementation will increase LDL-cholesterol concentrations (78). The genetic SNPs were not only related to circulating 25-hydroxyvitamin D but also plasma LDL-cholesterol and triglyceride levels. Pleiotropy-associated confounding cannot be completely ruled out (79).

The SNPs used in the previous two-stage and two-sample Mendelian randomization studies were mainly obtained from the SUNLIGHT consortium (study of underlying genetic determinants of Vitamin D and highly related traits) GWAS, which consisted of 31 cohorts from Europe, Canada, and the USA with atotal of 79,366 samples. The statistical effects of all SNPs were calculated by fixed-effect inverse-variance weighted meta-analysis. The joint test with multiple cohorts will easily induce an undetected bias due to the disparate measurements for 25(OH)D and the adjusted covariates. Meanwhile, we were unable to estimate the interactions between genetic variants and dietary intake of vitamin D as well as sunlight exposure as a source of vitamin D production in the skin. In the current study, the adequate instrumental SNPs involved in vitamin D synthesis (DHCR7/NADSYN1 and CYP2R1), transportation (GC), and degradation (CYP24A1), as well as novel vitamin D metabolism pathways, such as SEC23A (Sec23 homolog A, coat protein complex II component) and amidohydrolase domain containing 1 (AMDHD1), were recruited from the UK Biobank study with 401,460 white British participants. Moreover, the association between SNPs and 25(OH)D was estimated by linear mixed-model and the interactions of age, sex, and season with 25(OH)D were evaluated. The subjects in the GWAS analysis investigating the relationship between genetic loci and clinical features were of European and Caucasian descent, which is congruent with the population in UK Biobank. A comprehensive network of Mendelian randomization analysis, inverse-variance weighting (IVW), weighted median, weighted mode, and Mendelian randomization (MR)–Egger regression were performed to explore the causal effects and pleiotropy, as well as avoid bias. Mendelian randomization pleiotropy RESidual Sum and Outlier (MR-PRESSO) was conducted to uncover and exclude pleiotropic SNPs. The methods in our study provided a credible result for the large-scale study.

Here, several strengths of our study should be mentioned. First, the panoptic atlas of 25(OH)D in many clinical diseases and healthy traits were explored in this MR analysis, which will help us to have a more comprehensive understanding of the relationship between 25(OH)D and health. In addition, the bidirectional two-sample MR study was conducted to estimate the influence of diseases on 25(OH)D concentrations. Second, the SNP biomarkers and SNP estimates were obtained in mostly European studies, thus minimizing the possibility of population stratification bias. Third, the genetic SNPs for vitamin D were derived from a recent large-scale GWAS study (*n* = 443,734) rather than an earlier SUNLIGHT study, which may be more representative of the genetic instruments used to explore the genetic correlation of vitamin D.

However, there are some potential limitations worth noting in the current study.

Although we performed tests to prevent pleiotropy, some genetic SNPs are not only related to circulating 25-hydroxyvitamin D but also to traits, such as plasma LDL-cholesterol and triglyceride levels, and the potential pleiotropy cannot be definitively excluded. Moreover, the non-linear association between 25(OH)D and clinical outcomes could not be assessed by our two-sample MR analysis with a summary dataset. A non-linear MR analysis using raw data is needed in future. Meanwhile, 25(OH)D was recruited as the symbol of serum vitamin D concentration. In addition, the biological roles of active metabolite 1,25-dihydroxy vitamin D in clinical outcomes were not explored. Meanwhile, we still failed to study the biological roles of 24, 25-(OH)_2_-D_3_, 1, 24, 25-(OH)_3_-D_3_, and 25, 26-(OH)_2_-D_3_ metabolites and epimer of vitamin D in health (80). In addition, our results were not likely biased by pleiotropy due to the fact that 25OHD-associated genetic variants were not associated with other lifestyles, which influenced clinical outcomes, such as drinking, physical activity, or smoking. However, we cannot exclude the possibility that such an association may have a genetic basis rather than a causal relationship.

Hence, a large-scale genome-wide scan for genetic variants of vitamin D and further investigation to understand the potential role of vitamin D in the development of clinical outcomes are required. Meanwhile, a long-term and multicentric RCT study that can avoid the interference of numerous known and unpredictable confounders on the results, such as diet, exercise, sleep, geographic latitude, and climate, is seriously needed.

## Conclusion

Our study suggested that there was no evidence of the causal effect of 25(OH)D on numerous clinical diseases. Genetically decreased serum vitamin D was associated with estimated bone mineral density evaluated by ultrasound of the heel, plasma cholesterol, pulse pressure, and elevated lymphocyte count. Chronic kidney disease was inversely related to serum 25(OH)D concentration. The putative causal effects of vitamin D on multiple clinical diseases was not supported.

## Author contributions

K-qL: concept and design. J-jX: data collection, statistics, and writing the manuscript. X-bZ and TY: data collection. W-tT: data interpretation. K-qL and J-jX: study supervision. All authors critically revised the manuscript and approved the submitted version.

## Supplementary material

Supplementary Table 1 Characterics of 138 conditionally independent SNPs that reach genome-wide significance in the UKBB GWAS for 25OHD.

Supplementary Table 2 The effect estimates of the instrumental variables for 25(OH)D (exposure) and clinical diseases.

Supplementary Table 3 The effect estimates of the instrumental variables for clinical diseases (exposure) and 25(OH)D.

Supplementary Table 4 The effect estimates of the instrumental variables for 25(OH)D (exposure) and clinical traits.

Supplementary Table 5 Association of vitamin D with clinical diseases using other mendelian randomization studies in addition to inverse variance weighting.

Supplementary Table 6 Association of vitamin D with clinical traits using other mendelian randomization studies in addition to inverse variance weighting.

Supplementary Table 7 The International Classification of Diseases (ICD) codes for included clinical diseases.

Supplementary Figure 1 Assumptions of bidirectional Mendelian randomization analysis. **(a)** Main assumption of Mendelian randomization analysis. **(b)** Reversed assumption of Mendelian randomization analysis. (1) The genetic instrumental variables are strongly associated with exposures; (2) the genetic instrumental variables are not associated with any known or unmeasured confounders: influencing the association between genetic variants and outcomes; (3) the genetic variants are associated with outcomes only through exposures: variants causing significant effects on outcomes not through other pathways, no horizontal pleiotropy.

Supplementary Figure 2 Biological metabolic pathways of vitamin D.

Supplementary Figure 3 Scatter plot of the IVW MR study investigating the effect of 25(OH)D on clinical diseases. **(a)** Effect of vitamin D on chronic kidney disease. **(b)** Effect of vitamin D on stroke. **(c)** Effect of vitamin D on type-2 diabetes. **(d)** Effect of vitamin D on coronary artery disease. **(e)** Effect of vitamin D on coronary artery disease with diabetes. **(f)** Effect of vitamin D on Crohn's disease. **(g)** Effect of vitamin D on ulcerative colitis. **(h)** Effect of vitamin D on fracture. **(i)** Effect of vitamin D on rheumatoid arthritis. **(j)** Effect of vitamin D on Alzheimer's dementia. The *x*-axis represents the genetic association with 25OHD levels; the *y*-axis represents the genetic association with the risk of clinical diseases. The line represents the IVW method. 25OHD, 25-hydroxyvitamin D (vertical and horizontal blue lines around points show a 95% confidence interval for each polymorphism).

Supplementary Figure 4 Scatter plot of the IVW method in bidirectional Mendelian randomization analysis investigating the effect of clinical diseases on 25(OH)D. **(a)** Effect of chronic kidney disease on vitamin D. **(b)** Effect of stroke on vitamin D. **(c)** Effect of type-2 diabetes on vitamin D. **(d)** Effect of coronary artery disease on vitamin D. **(e)** Effect of coronary artery disease with diabetes on vitamin D. **(f)** Effect of Crohn's disease on vitamin D. **(g)** Effect of ulcerative colitis on vitamin D. **(h)** Effect of fracture on vitamin D. **(i)** Effect of rheumatoid arthritis on vitamin D. **(j)** Effect of Alzheimer's dementia on vitamin D. The *x*-axis represents the genetic association with the risk of clinical diseases; the *y*-axis represents the genetic association with 25OHD levels; The line represents the IVW method. 25OHD, 25-hydroxyvitamin D (vertical and horizontal blue lines around points show a 95% confidence interval for each polymorphism).
