# Supplementary figures and images for: Phenome-wide Mendelian randomization study evaluating the association of circulating vitamin D with complex diseases
Source: Front Nutr. 2023 Mar 29;10:1108477. doi: 10.3389/fnut.2023.1108477 (PMC10095159; doi:10.3389/fnut.2023.1108477)

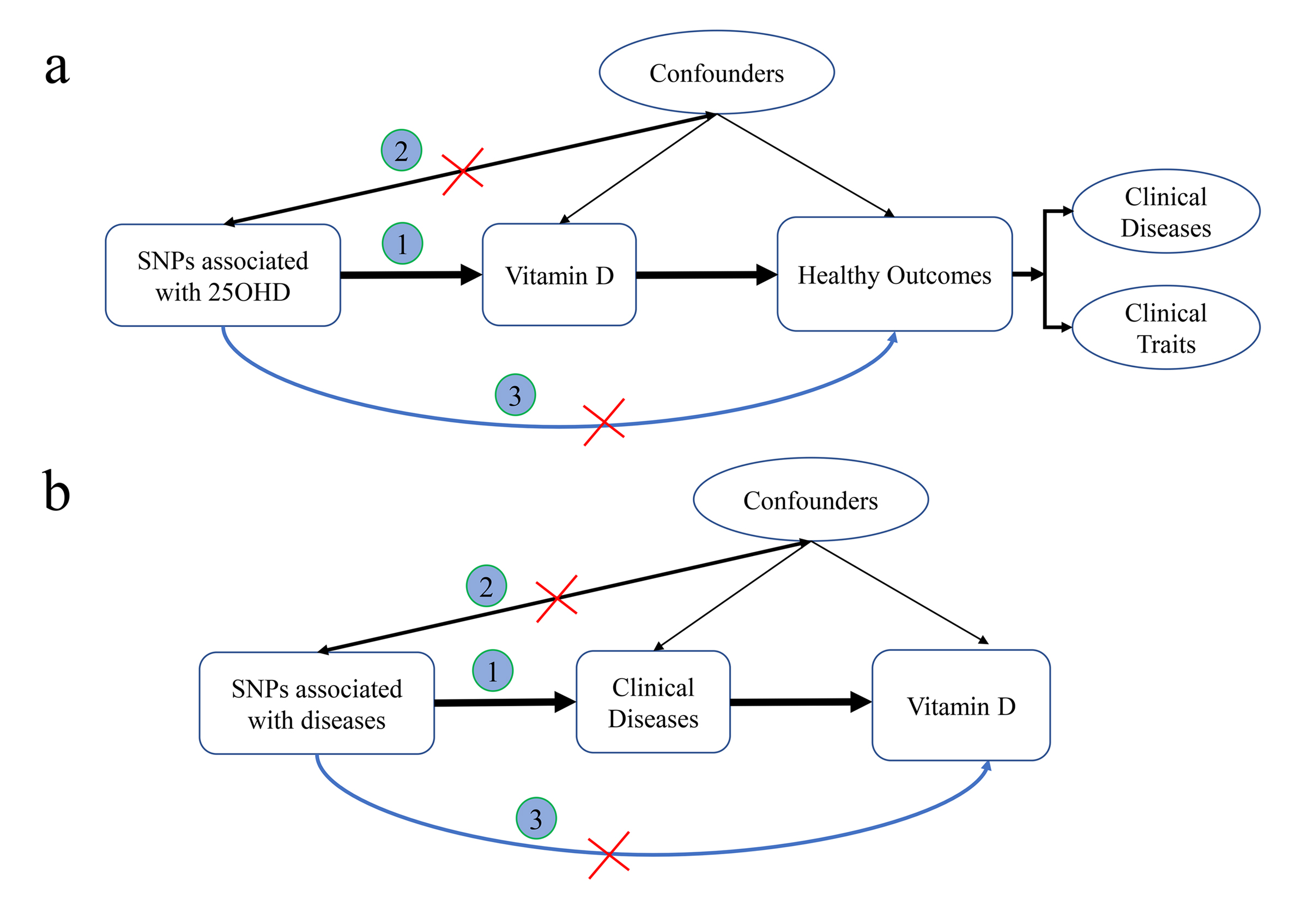

Supplement: Supplementary Figure 1 — Assumptions of bidirectional Mendelian randomization analysis. (a) Main assumption of Mendelian randomization analysis. (b) Reversed assumption of Mendelian randomization analysis. (1) The genetic instrumental variables are strongly associated with exposures; (2) the genetic instrumental variables are not associated with any known or unmeasured confounders: influencing the association between genetic variants and outcomes; (3) the genetic variants are associated with outcomes only through exposures: variants causing significant effects on outcomes not through other pathways, no horizontal pleiotropy. [file Image_1.JPEG]

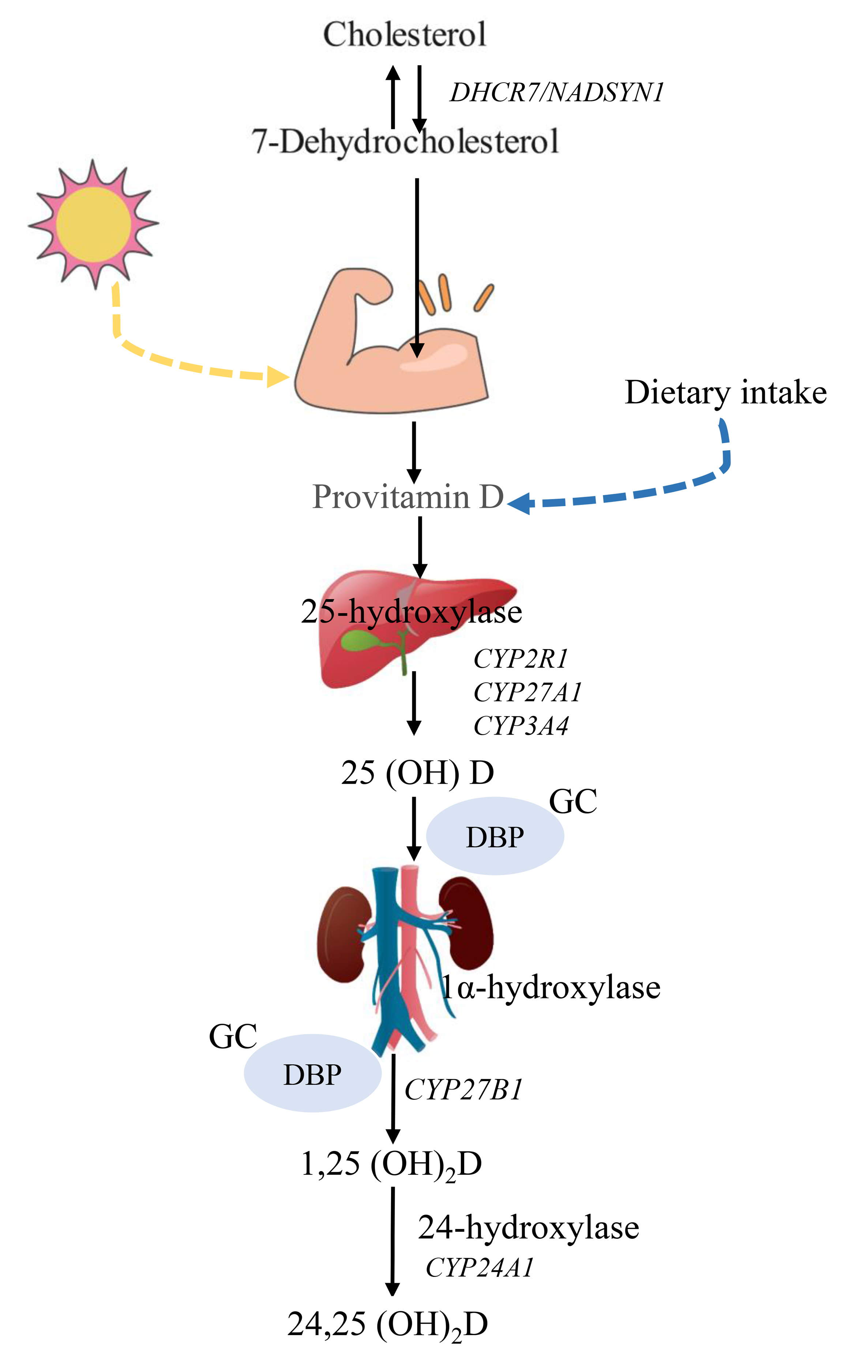

Supplement: Supplementary Figure 2 — Biological metabolic pathways of vitamin D. [file Image_2.JPEG]

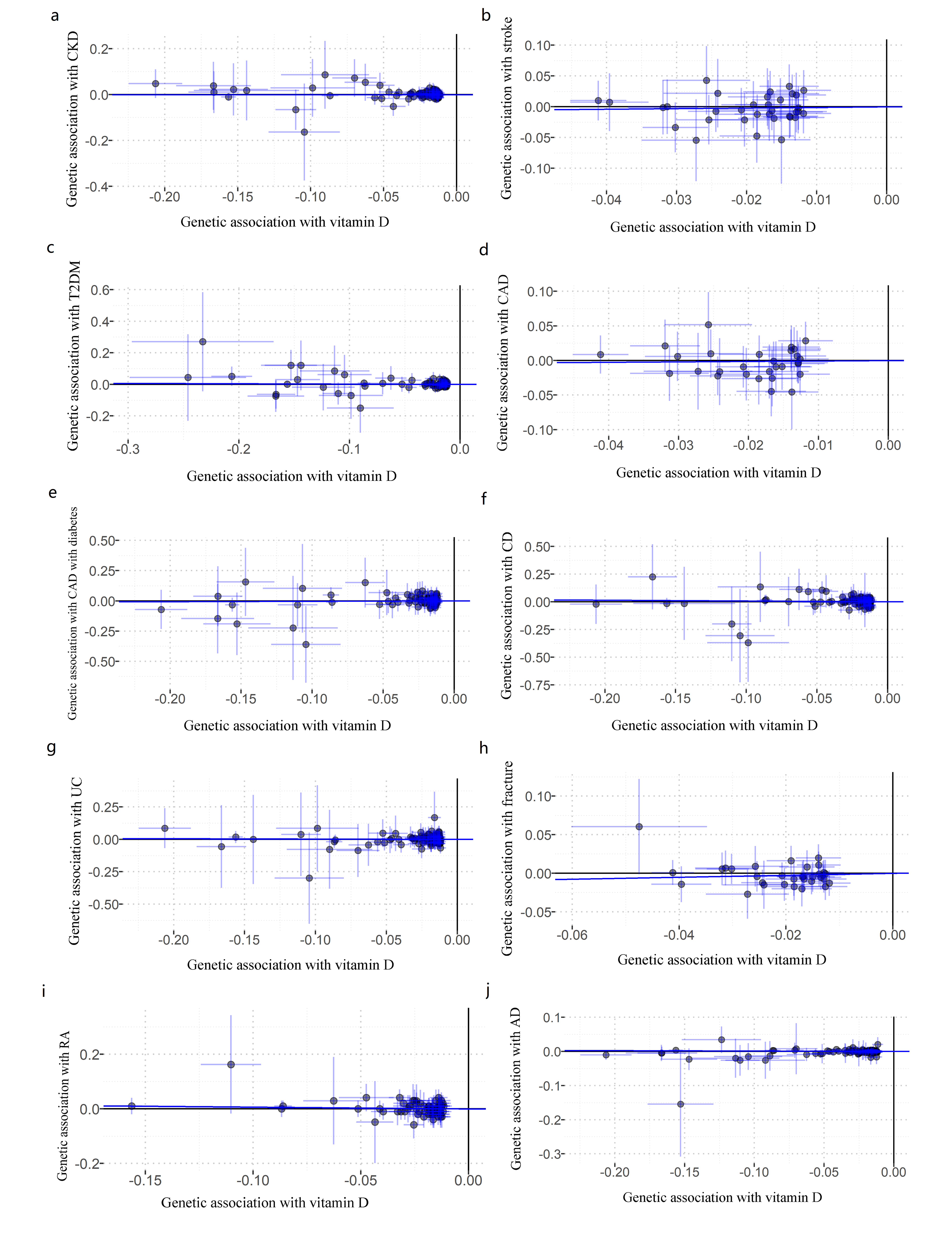

Supplement: Supplementary Figure 3 — Scatter plot of the IVW MR study investigating the effect of 25(OH)D on clinical diseases. (a) Effect of vitamin D on chronic kidney disease. (b) Effect of vitamin D on stroke. (c) Effect of vitamin D on type-2 diabetes. (d) Effect of vitamin D on coronary artery disease. (e) Effect of vitamin D on coronary artery disease with diabetes. (f) Effect of vitamin D on Crohn's disease. (g) Effect of vitamin D on ulcerative colitis. (h) Effect of vitamin D on fracture. (i) Effect of vitamin D on rheumatoid arthritis. (j) Effect of vitamin D on Alzheimer's dementia. The x-axis represents the genetic association with 25OHD levels; the y-axis represents the genetic association with the risk of clinical diseases. The line represents the IVW method. 25OHD, 25-hydroxyvitamin D (vertical and horizontal blue lines around points show a 95% confidence interval for each polymorphism). [file Image_3.JPEG]

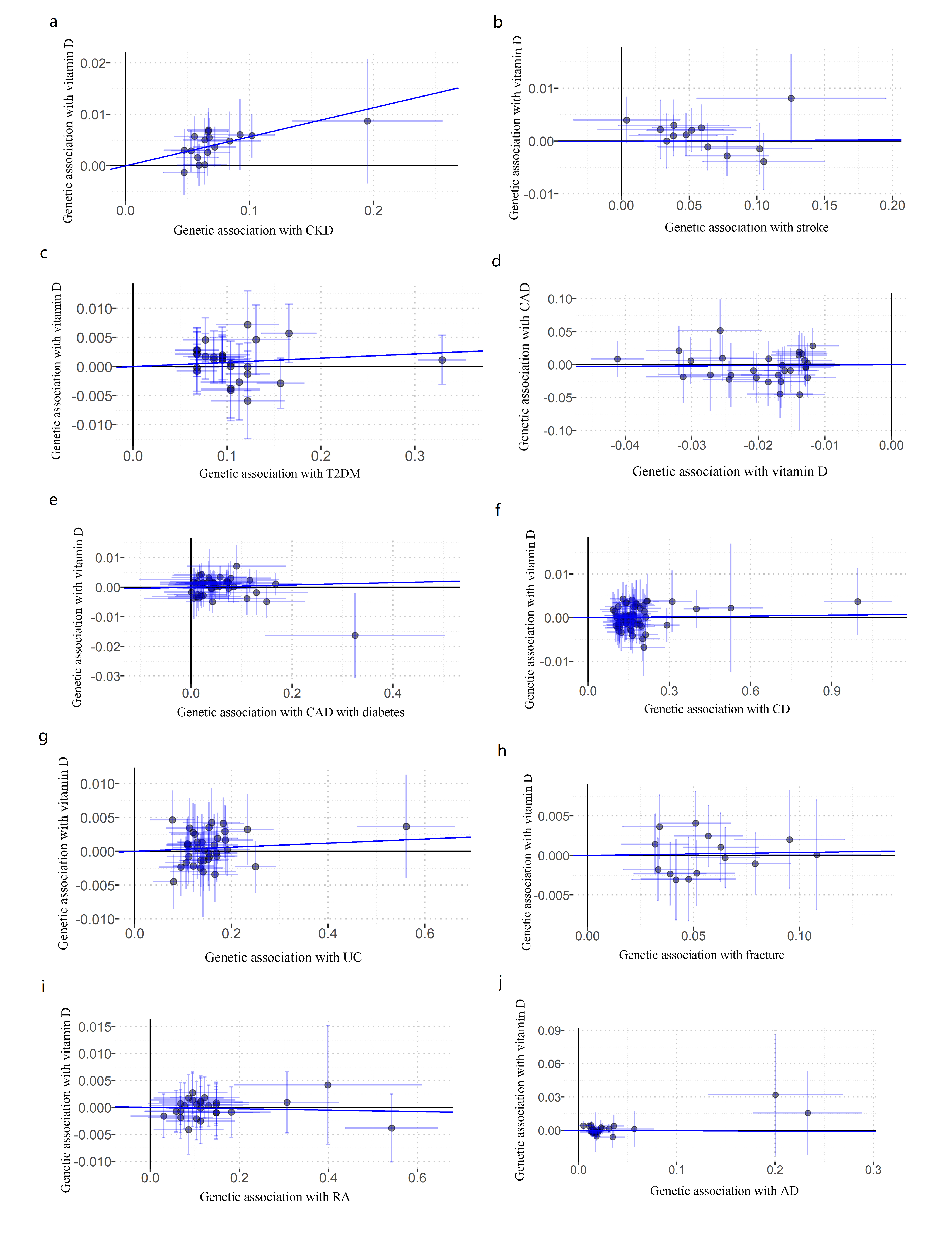

Supplement: Supplementary Figure 4 — Scatter plot of the IVW method in bidirectional Mendelian randomization analysis investigating the effect of clinical diseases on 25(OH)D. (a) Effect of chronic kidney disease on vitamin D. (b) Effect of stroke on vitamin D. (c) Effect of type-2 diabetes on vitamin D. (d) Effect of coronary artery disease on vitamin D. (e) Effect of coronary artery disease with diabetes on vitamin D. (f) Effect of Crohn's disease on vitamin D. (g) Effect of ulcerative colitis on vitamin D. (h) Effect of fracture on vitamin D. (i) Effect of rheumatoid arthritis on vitamin D. (j) Effect of Alzheimer's dementia on vitamin D. The x-axis represents the genetic association with the risk of clinical diseases; the y-axis represents the genetic association with 25OHD levels; The line represents the IVW method. 25OHD, 25-hydroxyvitamin D (vertical and horizontal blue lines around points show a 95% confidence interval for each polymorphism). [file Image_4.JPEG]
