# Supplementary material for: Phenome-wide Mendelian randomization study evaluating the association of circulating vitamin D with complex diseases
Source: Front Nutr. 2023 Mar 29;10:1108477. doi: 10.3389/fnut.2023.1108477 (PMC10095159; doi:10.3389/fnut.2023.1108477)
Supplement: Supplementary file 6 [file Data_Sheet_2.docx]

**Definition of health outcomes**

A participant was classified as having hypertension if she/he had SBP ≥140 mm Hg, or DBP ≥90 mm Hg, or was taking anti-hypertensive medication (ICD-10 codes: I10–I15)(Liu et al., 2016). The kidney function was evaluated by estimated glomerular filtration rate (eGFR), which was calculated by serum creatinine. The CKD (ICD-10 codes: N00–N08, N18, N17, N20–N23) was defined as an eGFR below 60 ml min^-1^ per 1.73 m^2^(Wuttke, Li, Li, Sieber, & Pattaro, 2019). Stroke (ICD-8 codes: 430–436, ICD-9 codes: 430–436, ICD-10 codes: I60-I64 and G45) was defined by WHO criteria as a sudden focal neurologic deficit of vascular origin, lasting more than 24 hours and confirmed by imaging such as computerized tomography (CT) and/or magnetic resonance imaging (MRI) brain scan(Malik et al., 2018). Patients with T2DM (ICD-10 codes: E11.901, E11.951-E11.955) were diagnosed by the criteria of fasting plasma glucose concentration ≥7.0 mmol/L or 2-h plasma glucose concentration ≥11.1 mmol/L, record of diabetes medication use(Mahajan et al., 2018). Cases of CAD (ICD-10 codes: I20-I25) were determined with a broad definition, including myocardial infarction (MI), acute coronary syndrome, chronic stable angina, and coronary artery stenosis 50%(Nikpay et al., 2015). The inflammatory bowel disease (IBD), mainly included ulcerative colitis (ICD-10 codes: K51.800, K51.851, K51.900-K51.904) and crohn's disease (ICD-10 codes: K50.000-k50.002, K50.100-k50.103), was defined by European Crohn’s and [Colitis](https://www.sciencedirect.com/topics/medicine-and-dentistry/colitis) Organization (ECCO) and American College of [Gastroenterology](https://www.sciencedirect.com/topics/medicine-and-dentistry/gastroenterology) (ECCO) according to [endoscopy](https://www.sciencedirect.com/topics/medicine-and-dentistry/endoscopy) gold standard(Anderson et al., 2011; Franke et al., 2010). Fracture (ICD-10 codes: M80.095, M80.295, M80.595, M80.895, M80.995, M84.491) cases were defined as those individuals (>18 years old) who had fractures at any skeletal site confirmed by medical, radiological, or questionnaire reports(Trajanoska et al., 2018). Fractures of the fingers, toes, and skull as well as high trauma fractures were excluded whenever possible. eBMD (g/cm^2^) was derived as a linear combination of speed of sound (SOS) and bone ultrasound attenuation (BUA) (eBMD = 0.002592 × (BUA + SOS) − 3.687)(Kemp et al., 2017). All RA cases fulfilled the 1987 criteria of the American College of Rheumatology for RA diagnosis, or were diagnosed as RA by a professional rheumatologist(Okada et al., 2014). Participants with a diagnosis of Alzheimer’s disease (diseases of the nervous system chapter in ICD-10; code G30) or dementia in Alzheimer’s disease (mental and behavioral disorders chapter in ICD-10; code F00) from any record of a hospital stay based on standardized examination of cognitive, functional and behavioral measures(Jansen et al., 2019).

**The network of mendelian randomization study**

In order to estimate the causal association of genetically determined vitamin D with healthy outcomes, five complementary MR methods were applied, including inverse-variance weighting (IVW)(Yavorska & Burgess, 2017), weighted median method(Bowden, Davey Smith, Haycock, & Burgess, 2016), mendelian randomization (MR)-Egger regression(Bowden, Davey Smith, & Burgess, 2015), weighted mode-based, and MR pleiotropy residual sum and outlier (MR-PRESSO)(Verbanck, Chen, Neale, & Do, 2018).

The IVW method was a weighted linear regression of the exposure effects against the outcome effects by inverse variance to set the intercept at zero. It obtained robust causal estimates without the directional pleiotropy. The weighted median method estimates some SNPs as invalid instruments, but still kept at least half are valid instruments for the causal effect estimate to be unbiased. The advantage of weighted median method was to improve the precision compared with MR Egger, but less accuracy of IVW. To test the directional (unbalanced) pleiotropy, we also used the MR-Egger regression method, which was based on the Egger test to detect the bias in meta-analysis. The MR-Egger regression achieved an instrument exposure effect against its effect upon the outcome, while its intercept was free (non-zero) estimation compared with IVW method. If there was a statistically significant intercept term in MR Egger regression, it implicated a presence of horizontal pleiotropy, which meant the effects of the SNPs on the outcome not mediated by the exposure. The mode-based method was clustered the similar causal effects into different groups, the unbiased causal effect estimate was derived from the group of the largest number of SNPs. The weighted mode method calculated every SNP’s contribution to the clustering using the inverse variance of their outcome effects. The MR-PRESSO was a slope of the line regression of the variants’ effects on exposure against the same variants’ effects on the outcome, its important function was whether there was the horizontal pleiotropic in different instruments MR test by using the observed and expected distributions of the tested variants. If there was a horizontal pleiotropic, the MR-PRESSO test adjusted horizontal pleiotropy via the function of outlier removal. Even more, it showed the causal effects before and after adjusted for removal outliers. Generally, we used the inverse variance-weighted, MR Egger and MR-PRESSO as supplements to obtain the causal effects. We implemented the Egger intercept test and MR-PRESSO to evaluate the horizontal pleiotropy in heterogeneity tests between individual genetic variants. Furthermore, we also used the modified Cochran’s Q statistic to evaluate the instrument strength and validity instead of measurement error in the SNP-exposure effect(Burgess, Foley, Allara, Staley, & Howson, 2020).

Anderson, C. A., Boucher, G., Lees, C. W., Franke, A., D'Amato, M., Taylor, K. D., . . . Rioux, J. D. (2011). Meta-analysis identifies 29 additional ulcerative colitis risk loci, increasing the number of confirmed associations to 47. *Nat Genet, 43*(3), 246-252. <https://doi.org/10.1038/ng.764>.

Bowden, J., Davey Smith, G., & Burgess, S. (2015). Mendelian randomization with invalid instruments: effect estimation and bias detection through Egger regression. *Int J Epidemiol, 44*(2), 512-525. <https://doi.org/10.1093/ije/dyv080>.

Bowden, J., Davey Smith, G., Haycock, P. C., & Burgess, S. (2016). Consistent Estimation in Mendelian Randomization with Some Invalid Instruments Using a Weighted Median Estimator. *Genet Epidemiol, 40*(4), 304-314. <https://doi.org/10.1002/gepi.21965>.

Burgess, S., Foley, C. N., Allara, E., Staley, J. R., & Howson, J. M. M. (2020). A robust and efficient method for Mendelian randomization with hundreds of genetic variants. *Nat Commun, 11*(1), 376. <https://doi.org/10.1038/s41467-019-14156-4>.

Franke, A., McGovern, D. P. B., Barrett, J. C., Wang, K., Radford-Smith, G. L., Ahmad, T., . . . Parkes, M. (2010). Genome-wide meta-analysis increases to 71 the number of confirmed Crohn's disease susceptibility loci. *Nature Genetics, 42*(12), 1118-1125. <https://doi.org/10.1038/ng.717>.

Jansen, I. E., Savage, J. E., Watanabe, K., Bryois, J., Williams, D. M., Steinberg, S., . . . Posthuma, D. (2019). Genome-wide meta-analysis identifies new loci and functional pathways influencing Alzheimer's disease risk. *Nat Genet, 51*(3), 404-413. <https://doi.org/10.1038/s41588-018-0311-9>.

Kemp, J. P., Morris, J. A., Medina-Gomez, C., Forgetta, V., Warrington, N. M., Youlten, S. E., . . . Evans, D. M. (2017). Identification of 153 new loci associated with heel bone mineral density and functional involvement of GPC6 in osteoporosis. *Nat Genet, 49*(10), 1468-1475. <https://doi.org/10.1038/ng.3949>.

Liu, C., Kraja, A. T., Smith, J. A., Brody, J. A., Franceschini, N., Bis, J. C., . . . Chasman, D. I. (2016). Meta-analysis identifies common and rare variants influencing blood pressure and overlapping with metabolic trait loci. *Nat Genet, 48*(10), 1162-1170. <https://doi.org/10.1038/ng.3660>.

Mahajan, A., Taliun, D., Thurner, M., Robertson, N. R., Torres, J. M., Rayner, N. W., . . . McCarthy, M. I. (2018). Fine-mapping type 2 diabetes loci to single-variant resolution using high-density imputation and islet-specific epigenome maps. *Nat Genet, 50*(11), 1505-1513. <https://doi.org/10.1038/s41588-018-0241-6>.

Malik, R., Chauhan, G., Traylor, M., Sargurupremraj, M., Okada, Y., Mishra, A., . . . Dichgans, M. (2018). Multiancestry genome-wide association study of 520,000 subjects identifies 32 loci associated with stroke and stroke subtypes. *Nat Genet, 50*(4), 524-537. <https://doi.org/10.1038/s41588-018-0058-3>.

Nikpay, M., Goel, A., Won, H. H., Hall, L. M., Willenborg, C., Kanoni, S., . . . Farrall, M. (2015). A comprehensive 1,000 Genomes-based genome-wide association meta-analysis of coronary artery disease. *Nat Genet, 47*(10), 1121-1130. <https://doi.org/10.1038/ng.3396>.

Okada, Y., Wu, D., Trynka, G., Raj, T., Terao, C., Ikari, K., . . . Plenge, R. M. (2014). Genetics of rheumatoid arthritis contributes to biology and drug discovery. *Nature, 506*(7488), 376-381. <https://doi.org/10.1038/nature12873>.

Trajanoska, K., Morris, J. A., Oei, L., Zheng, H. F., Evans, D. M., Kiel, D. P., . . . the 23andMe research, t. (2018). Assessment of the genetic and clinical determinants of fracture risk: genome wide association and mendelian randomisation study. *BMJ, 362*, k3225. <https://doi.org/10.1136/bmj.k3225>.

Verbanck, M., Chen, C. Y., Neale, B., & Do, R. (2018). Detection of widespread horizontal pleiotropy in causal relationships inferred from Mendelian randomization between complex traits and diseases. *Nat Genet, 50*(5), 693-698. <https://doi.org/10.1038/s41588-018-0099-7>.

Wuttke, M., Li, Y., Li, M., Sieber, K. B., & Pattaro, C. (2019). A catalog of genetic loci associated with kidney function from analyses of a million individuals. *Nature Genetics, 51*(6), 957–972. <https://doi.org/10.1038/s41588-019-0407-x>.

Yavorska, O. O., & Burgess, S. (2017). MendelianRandomization: an R package for performing Mendelian randomization analyses using summarized data. *Int J Epidemiol, 46*(6), 1734-1739. <https://doi.org/10.1093/ije/dyx034>.
